# Rapid Response System Improves Sepsis Bundle Compliances and Survival in Hospital Wards for 10 Years

**DOI:** 10.3390/jcm10184244

**Published:** 2021-09-18

**Authors:** Sunhui Choi, Jeongsuk Son, Dong Kyu Oh, Jin Won Huh, Chae-Man Lim, Sang-Bum Hong

**Affiliations:** 1 Medical Emergency Team, Asan Medical Center, Seoul 05505, Korea; sunhui.choi@amc.seoul.kr (S.C.); js_son@amc.seoul.kr (J.S.); 2 Asan Medical Center, Department of Pulmonary and Critical Care Medicine, University of Ulsan College of Medicine, Seoul 05505, Korea; synthesis83@hanmail.net (D.K.O.); jwhuh@amc.seoul.kr (J.W.H.); cmlim@amc.seoul.kr (C.-M.L.)

**Keywords:** sepsis, shock, septic, rapid response systems, hospital-onset sepsis, hospital rapid response team

## Abstract

Background: Hospitalized patients can develop septic shock at any time. Therefore, it is important to identify septic patients in hospital wards and rapidly perform the optimal treatment. Although the sepsis bundle has already been reported to improve survival rates, the controversy over evidence of the effect of in-hospital sepsis continues to exist. We aimed to estimate the outcomes and bundle compliance of patients with septic shock in hospital wards managed through the rapid response system (RRS). Methods: A retrospective cohort study of 976 patients with septic shock managed through the RRS at an academic, tertiary care hospital in Korea from 2008 to 2017. Results: Of the 976 enrolled patients, the compliance of each sepsis bundle was high (80.8–100.0%), but the overall success rate of the bundle was low (58.3%). The compliance rate for achieving the overall sepsis bundle increased from 26.5% to 70.0%, and the 28-day mortality continuously decreased from 50% to 32.1% over 10 years. We analyzed the two groups according to whether they completed the overall sepsis bundle or not. Of the 976 enrolled patients, 569 (58.3%) sepsis bundles were completed, whereas 407 (41.7%) were incomplete. The complete bundle group showed lower 28-day mortality than the incomplete bundle group (37.1% vs. 53.6%, *p* < 0.001). In the multivariate multiple logistic regression model, the 28-day mortality was significantly associated with the complete bundle (adjusted odds ratio (OR), 0.61; 95% confidence intervals (CI), 0.40–0.91; *p* = 0.017). The obtaining of blood cultures (adjusted OR, 0.45; 95% CI, 0.33–0.63; *p* < 0.001) and lactate re-measurement (adjusted OR, 0.69; 95% CI, 0.50–0.95; *p* = 0.024) in each component of the sepsis bundle were associated with the 28-day mortality. Conclusions: The rapid response system provides improving sepsis bundle compliances and survival in patients with septic shock in hospital wards.

## 1. Introduction

Sepsis and septic shock present in more than 50% of adult hospitalizations ending in death or terminal discharge to a hospice. Since sepsis and septic shock patients require invasive treatment and intensive hemodynamic monitoring, most of them need to be treated in intensive care units (ICUs) [1]. Rescuing these patients requires early, aggressive, and appropriate fluid resuscitation, control of the source of infection, and antimicrobial therapy [2,3]. The surviving sepsis campaign (SSC) has been providing guidelines for the treatment of sepsis since 2002 and has highlighted sepsis bundles [4,5].

According to the 7.5-year study of the SSC, the rate of mortality due to sepsis in hospital wards was 40.3%, which is much higher than the 26.0% in emergency departments. This was similar to the mortality rate (44.2%) among patients in the ICU [6]. In addition, the cost of hospitalization for patients with hospital-onset sepsis is five times that of patients with community-onset sepsis [7]. According to a recently reported large cohort study, the complete bundle of sepsis was not associated with outcome in hospital-onset sepsis, and only early broad-spectrum intravenous antibiotic treatment among the bundle elements was associated with reduced mortality [8]. The guidelines for the management of sepsis focus on early detection and treatment of community-onset sepsis in emergency rooms. However, other strategies may be required to detect and treat hospital-onset sepsis, i.e., sepsis occurring during hospitalization. Management strategies to improve the recognition and management of hospital-onset sepsis are geared to the use of rapid response systems (RRS) [7]. Although sepsis bundles have already been reported to improve survival rates, the controversy over evidence of the effect on in-hospital sepsis continues to exist [8,9,10]. We aimed to estimate the outcomes and bundle compliance of patients with septic shock in hospital wards managed through the RRS.

## 2. Materials and Methods

### 2.1. Study Design and Ethical Approval

We performed a retrospective cohort study to estimate the outcomes and bundle compliance of patients with septic shock managed through the RRS in an academic, tertiary care hospital equipped with approximately 2700 beds (Asan Medical Center, Seoul, Korea). We collected and analyzed clinical data between March 2008 and December 2017.

The experimental plan used for this study received approval from our institutional review board (IRB No. 2020–0286) and was conducted in accordance with the Korea Food and Drug Administration and the International Conference on Harmonization of Good Clinical Practice guidelines.

### 2.2. Data Collection and Definitions

The following data on sepsis patients triggering RRS activation were received from the RRS registry and electronic medical records. During the study period, laboratory findings, comorbidities, departments, sources of infection, and blood cultures were recorded to identify the characteristics of patients with sepsis in hospital wards. Furthermore, to confirm sepsis management in hospital wards, the amounts of fluids used for resuscitation, application of vasopressors, use of point of care ultrasound, placement of central and arterial catheters, and source control were assessed.

The severity of illness was assessed using the Sequential (Sepsis-related) Organ Function Assessment (SOFA) score, which was measured within 6 h after RRS activation. Time zero was defined as the time reported by the attending staff for RRS activation or the time of recognition of sepsis as reported by the RRS staff through the RRS screening system.

Data were collected on each element of the SSC sepsis bundle used to perform treatment for patients [11]. The three-hour bundle for sepsis comprises four elements; the measurement of the serum lactate level, the acquisition of blood cultures prior to antibiotic therapy, the administration of broad-spectrum antibiotics, and the administration of IV fluids. The six-hour bundle for sepsis comprises two elements; administration of vasopressors and the re-measurement of serum lactate levels if the initial levels were elevated.

The primary outcome of this study was the 28-day mortality rate according to the overall completion rate of the sepsis bundle. The secondary outcome was risk factors for 28-day mortality.

### 2.3. Role of Rapid Response Systems (RRS) in Sepsis

Our hospital has had the RRS since 2008. The RRS operated for 24 h a day, 7 days a week and the team consist of intensivists and dedicated clinical nurse specialists. The RRS was activated if a patient is identified by automated screening system consisting of vital sign parameter or early warning score or laboratory measurement based on the electronic medical record (EMR) or activated through direct phone call by ward staff or nurses.

We detected patients early through the screening system and performed sepsis assessment, management and inter-professional collaboration with ward staff and nurses. We performed invasive procedures or addition managements through the evaluation of fluid volume status. In addition, the RRS provided a staff education focus on early identification and giving timely treatments and care bundles for sepsis.

### 2.4. Statistical Analysis

Statistical analysis of the collected data was performed using IBM SPSS Statistics version 21 (IBM Corp., Armonk, NY, USA).

Data are presented as mean ± SD for continuous variables, including age, SOFA score, and laboratory data, and as frequencies (%) for categorical variables, including sex, type of sepsis, and source of infection. Statistical analysis was performed using the *t*-test for continuous variables and the χ^2^ or Fisher’s exact test for categorical variables as appropriate.

The associations between bundle compliance and 28-day mortality were analyzed using logistic regression. We adjusted for age, sex, call type, completion of all bundles, use of ventilator support, positivity of blood cultures, initial serum lactate level, follow-up serum lactate level, C-reactive protein level, procalcitonin level, total volume of fluid administered over 6 h, use of inotropics, transfusion of red blood cells, use of steroids, source control, and SOFA score. The results are presented as odds ratios (ORs) with 95% confidence intervals (CIs). *p*-values of less than 0.05 were considered statistically significant.

## 3. Results

**Figure 1:**
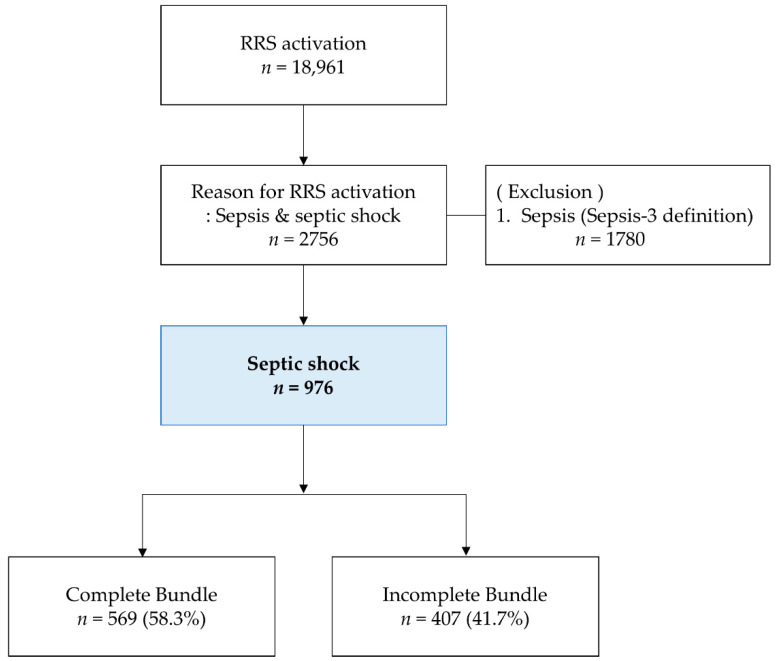
Flow diagram of the process of selection of study patients.

Figure 1 shows the flow diagram for sepsis management through the RRS. We managed sepsis through the RRS during the study period. Of the 976 enrolled patients with septic shock, the compliances of each sepsis bundle were high (80.8%–100.0%); however, the overall completion rate of the bundles was shown to be low (58.3%; Table 1 and Table S1).

**Figure 2:**
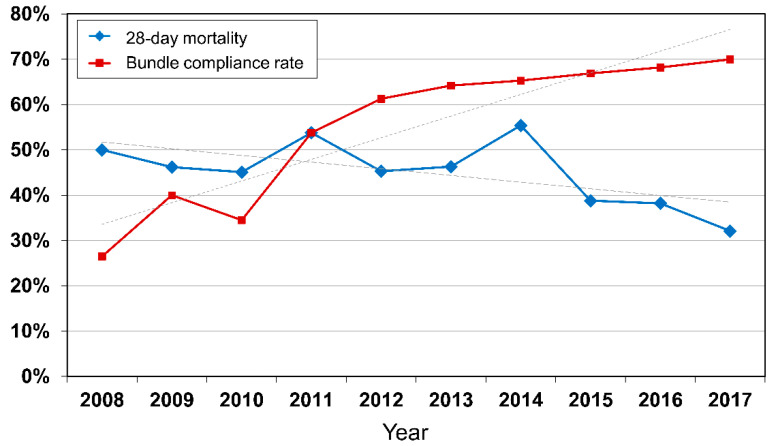
Compliance rate for achieving overall sepsis bundle and 28-day mortality rate of septic shock.

Figure 2 shows the compliance rate for achieving overall sepsis bundles and the 28-day mortality rate for patients with septic shock who were referred to the RRS from March 2008 to December 2017. The compliance rate for achieving the overall sepsis bundle increased and the 28-day mortality continuously decreased over 10 years.

We analyzed the two groups according to whether or not they completed the overall sepsis bundle. Of the 976 enrolled patients, 569 (58.3%) completed all sepsis bundles, while 407 (41.7%) did not complete them. The mean age, sex, co-morbidity, and department did not differ significantly between the two groups. The SOFA score was significantly higher in the incomplete bundle group than in the complete bundle group (10.6 ± 3.5 vs. 11.1 ± 3.7, *p* = 0.029). C-reactive protein levels were significantly higher in the incomplete bundle group than in the complete bundle group (12.20 ± 9.52 vs. 13.38 ± 10.37, *p* = 0.002). Serum creatinine levels were significantly higher in the incomplete bundle group than in the complete bundle group (1.68 ± 1.32 vs. 1.85 ± 1.59, *p* = 0.002; Table S2).

The initial management of shock within 6 h differed significantly in the two study groups. The patients in the complete bundle group more frequently required fluid resuscitation (2.34 ± 1.26 L vs. 1.77 ± 1.36 L, *p* < 0.001), vasopressin (40.4% vs. 22.4%, *p* < 0.001), epinephrine (12.0% vs. 6.9%, *p* = 0.005), use of point of care ultrasound (45.0% vs. 25.3%, *p* < 0.001), arterial catheters (72.6% vs. 60.9%, *p* < 0.001), and source control (20.2% vs. 13.3%, *p* = 0.003) than those in the incomplete bundle group; however, the patients in the incomplete bundle group more frequently required dopamine (4.0% vs. 6.6%, *p* = 0.049), and mechanical ventilation (32.3% vs. 39.1%, *p* = 0.018; Table 2) than those in the complete bundle group.

A total of 578 patients (59.2%) were transferred to the ICU, with the complete bundle group accounting for a significantly higher proportion than the incomplete bundle group (62.4% vs. 54.8%, *p* = 0.010). The overall 28-day mortality rate was 44.0%, with the rate being significantly lower in the complete bundle group than in the incomplete bundle group (37.1% vs. 53.6%, *p* <0.001), and similar tendencies being observed for the in-hospital mortality rate (42.4% vs. 57.0%, *p* < 0.001; Table 3).

In the multiple multivariate logistic regression model, 28-day mortality was significantly associated with all bundle completion (adjusted odds ratio, 0.61; 95% CI, 0.40–0.91; *p* = 0.017), re-measured serum lactate level (adjusted odds ratio, 1.20; 95% CI, 1.12–1.29; *p* < 0.001), C-reactive protein (adjusted odds ratio, 1.04; 95% CI, 1.02–1.06; *p* < 0.001), insertion of arterial catheter (adjusted odds ratio, 0.59; 95% CI, 0.38–0.91; *p* = 0.018), source control (adjusted odds ratio, 0.50; 95% CI, 0.30–0.84; *p* = 0.008), and SOFA score (adjusted odds ratio, 1.33; 95% CI, 1.23–1.44; *p* < 0.001, Table 4).

**Figure 3:**
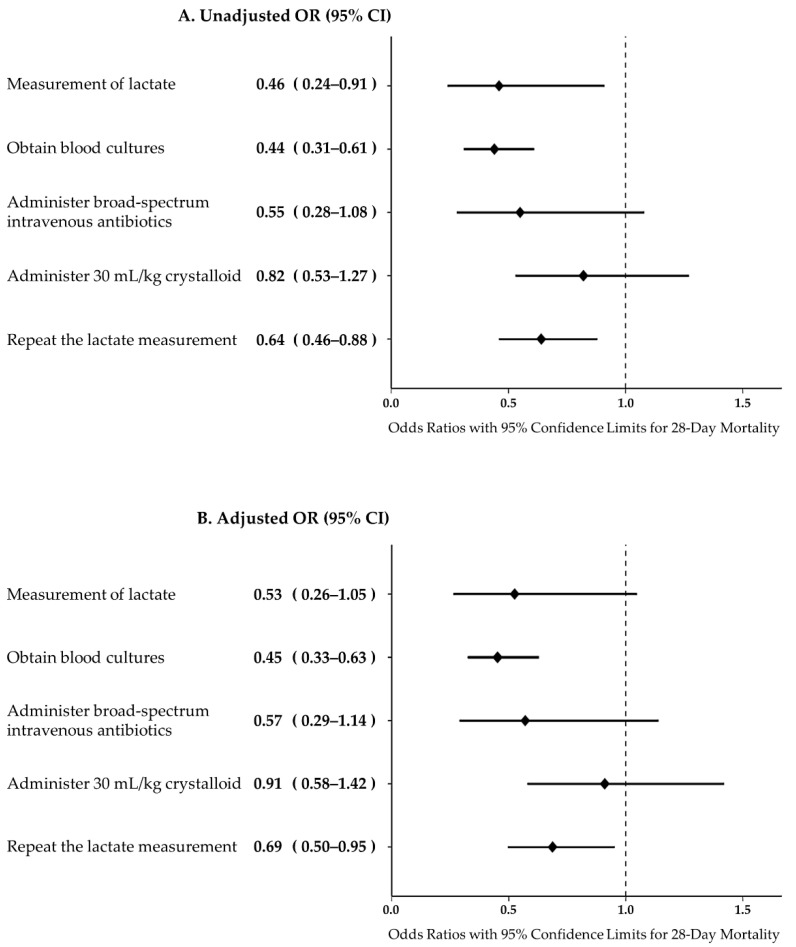
Multiple multivariate logistic regression model for bundle components with 28-day mortality in septic shock patients; OR, odds ratio, CI, confidence interval.

The obtaining of blood cultures (adjusted odds ratio, 0.45; 95% CI, 0.33–0.63; *p* < 0.001) and the lactate level re-measurement (adjusted odds ratio, 0.69; 95% CI, 0.50–0.95; *p* = 0.024) in each component of the sepsis bundle were associated with the risk of 28-day mortality (Figure 3).

## 4. Discussion

In this study, we showed that RRSs have improved compliance with sepsis bundles for 10 years, and compliance of sepsis bundles was associated with reduced 28-day mortality in patients with septic shock in hospital wards. The compliance rate of the 3/6-h bundle increased from 26.5% to 70.0% and the 28-day mortality decreased from 50.0% to 32.1% over 10 years. It was also confirmed that bundle completion, re-measured serum lactate levels, C-reactive protein levels, insertion of arterial catheter, source control, and SOFA score were associated with 28-day mortality. In addition, obtaining of blood cultures and lactate re-measurement among the detailed elements of the sepsis bundle were classified as factors associated with 28-day mortality.

One observational study conducted over 7.5 years in SSC reported a 3–5% decrease in in-hospital mortality for every 10% increase in bundle compliance [6]. In addition, a large-scale retrospective study reported that a more rapid completion of the 3 h bundle and the administration of broad-spectrum antibiotics were associated with higher in-hospital mortality among patients with severe sepsis and septic shock in the emergency department [3]. Recently, the sepsis bundle core performance measure was rolled out in the centers for medicare and medicaid services inpatient quality reporting program beginning in 2015, with the aim of facilitating timely, high-quality sepsis care, and many studies on hospital-onset sepsis have been reported [8,12,13]. According to a recently reported large cohort study, the compliance rate for bundles in community-onset sepsis was 40.1%, whereas it was only 12.2% in hospital-onset sepsis. The complete bundle was not associated with outcome in hospital-onset sepsis, and only early broad-spectrum intravenous antibiotic treatment among the bundle elements was associated with outcome [8]. In addition, a retrospective cohort study demonstrated that although sepsis bundle failure was not associated with mortality, the overall compliance rate for bundles was only 33.0%, and this study included only 9% of hospital-onset sepsis cases [14]. There is still controversy over whether or not the compliance of bundle reduces mortality due to in-hospital sepsis. Our study demonstrates that bundle compliance is associated with mortality in hospitalized patients with septic shock. A possible explanation for this might be that the overall bundle compliance rate in our study was found to be 58.3%, which is higher than that of previously reported studies on hospital-onset sepsis. Considering the high rate of bundle compliance in the emergency department, the difference in bundle compliance rate might have been an influence.

In this study, we analyzed the factors associated with mortality among the detailed elements of sepsis bundles, which, contrary to expectations, the obtaining of blood cultures was classified as one of the factors associated with 28-day mortality. There was no significant association between the administration of broad-spectrum antibiotics and outcome. A retrospective review study demonstrated that the time required for the completion of a 3-h bundle and the administration of broad-spectrum antibiotics were associated with higher in-hospital mortality among patients with severe sepsis and septic shock in the emergency department [3]. In contrast to patients staying in the emergency room, patients who are hospitalized often become worse with sepsis while they are already infected, and they are often already administering antibiotics. In this study, antibiotic administration in septic shock was performed in 96.3% of cases. However, no analysis on the appropriateness, addition, or change of antibiotics was conducted. In general, at the onset of sepsis, blood cultures should be obtained prior to antibiotic administration, as the obtaining of blood cultures during antibiotic therapy is associated with the loss of clinically relevant pathogen identification [15]. It might be important to determine whether the deterioration of patients already receiving antibiotic therapy for septic shock is a deterioration of the existing infection status, a mutation of the pathogens into antibiotic-resistant bacteria, or a new infection. Therefore, further research on whether identifying the causative bacteria needs to be carried out by conducting culture tests to identify new sources of infection rather than using antibiotics will lead to the optimization of antibiotics and improved survival rates.

In our analysis, another detailed element of the sepsis bundle associated with mortality was lactate re-measurement and not initial lactate measurement. A possible explanation for this might be that that lactate clearance is associated with the outcome rather than the initial measurement of the level of lactate, reflecting tissue perfusion. This differs from the result of a previous study that reported that early lactate measurement among bundle items was associated with mortality in community-onset sepsis; however, lactate re-measurement was not included in the statistics and analysis in that study [8]. In addition, in a retrospective cohort study conducted at seven U.S. hospitals, the most common reasons for failure were not measuring initial or repeat lactate levels but noncompliant care was not associated with higher mortality [14]. A meta-analysis of four small randomized trials reported that the use of lactate clearance as an umpire to guide early therapy is associated with a reduction in the risk of death in adult patients with sepsis [16]. Early lactate clearance-guided therapy was found to be effective in terms of significantly reducing mortality, shortening the length of ICU stay and duration mechanical ventilation, and reducing the Acute Physiology and Chronic Health Evaluation-II (APACHE-II) score [17]. Lactate re-measurement within 2–4 h is easy to forget because it is not included in the 1-hour bundle of sepsis; however, this study suggests that it is necessary to check the repeated lactate level and perform additional treatment according to the lactate clearance.

This study may support the hypothesis that the overall bundle compliance rate of septic shock continues to increase as the RRS continues to operate and matures. Although sepsis bundles consist of many simple elements, it is difficult to achieve all items in a fixed time. In a previous study, 1647 out of 4108 patients with community-onset sepsis achieved an overall compliance rate of 40.0%, while only 281 of 2296 patients with hospital-onset sepsis achieved a bundle, showing an overall compliance rate of 12.2% [8]. Rhee et al. reported that the cases in which sepsis bundles failed were more likely to have septic shock, hospital-onset sepsis, vague rather than explicit infectious symptoms, and non-pulmonary infections compared to cases that passed [14]. Although for sepsis third definition criteria have been announced, the diagnosis of sepsis is still equivocal, and clinical judgment of whether or not there is an infection is complicated [18]. In addition, since vital signs are intermittently measured every 8–12 h and laboratory tests are not routinely performed in hospital wards, the early detection of sepsis there is more difficult than it is in emergency wards or ICUs. SSC international guidelines have emphasized that hospitals should have a system for sepsis screening [4]; however, existing studies lack analysis of systems. The RRS is equipped with a screening system that uses early warning scores or physiological parameters that include hemodynamic indicators; as such, it is used as an important tool for the early detection of sepsis patients. Our RRS is operating a 24 h EMR-based screening system, and we observed that 57.5% of patients with septic shock were triggered to RRS by the EMR-based screening system. Given that the detection of sepsis should lead to immediate treatment, these findings support the RRS as a sepsis team plays a role in the early detection of sepsis and its early management in hospital wards.

This study has some strengths, among which is the fact that we have identified treatment performances and outcomes for septic shock patients in hospital wards. However, there are several limitations that hinder the generalizability of our results. First, this study was conducted on septic shock patients activated to the RRS due to clinical deterioration. It is hard to verify whether the delayed recognition or delayed management of sepsis in the ward caused the sepsis to deteriorate before the RRS was activated. The SSC recommends the performance of the bundle of sepsis if sepsis is suspected, which should be carried out before resorting to RRS, which is difficult to confirm through a record review due to the uncertainty of time zero and is beyond the scope of this study. However, questions about the initial treatment delay still stand. Second, the overall compliance rate in this study was higher than those of other studies because the time zero was clearly based on the RRS contact time assessed with sepsis. The criterion of “time zero” should be considered as the reason why the bundle compliance rate differs in many studies [19,20,21]. It is difficult to objectively define the timing of sepsis recognition presented in the SSC, and the definitions of time zero in existing retrospective studies vary. In addition, it is difficult to determine whether the subtle change worsens sepsis, as patients who are hospitalized are already undergoing acute treatment, unlike those in emergency rooms. Therefore, setting the time zero of sepsis treatment also often depends on the clinical decision of the physician. This suggests the need for objective criteria for a clear time zero. Third, we evaluated the administration of antibiotics but not antibiotic adequacy or escalation to guidelines. In hospital sepsis, antibiotics are often already being administered, so clear guidelines for proper antibiotics to be administered or escalated are required. Lastly, this study is a retrospective observational study in a single tertiary referral center. We included patients according to the sepsis-3 definition, but some patients might be omitted. In addition, as hospitals operate various types of RRS in terms of staff members or operating time, depending on how RRS operates can affect the different results.

## 5. Conclusions

Surveillance, early management and complete bundle in patients with sepsis by RRS was improved through the years and the compliance of sepsis bundles was associated with reduced 28-day mortality in patients with septic shock in hospital wards. In addition, obtaining blood cultures and lactate re-measurements, among the detailed elements of sepsis bundles, were significantly associated with the 28-day mortality rate.

## Figures and Tables

**Table 1:** The 3 h and 6 h elements and compliance of sepsis bundle for patients with septic shock.

Variables	All (*n* = 976)
Measurement of lactate/3 h	939 (96.2)
Obtain blood cultures/3 h	791 (81.0)
Administer broad-spectrum intravenous antibiotics/3 h	940 (96.3)
Administer 30 mL/kg crystalloid/3 h	885 (90.7)
Application of vasopressors/6 h	976 (100.0)
Repeat the lactate measurement/6 h	789 (80.8)
Complete bundle. overall	569 (58.3)

Data are presented as *n* (%).

**Table 2:** Management of shock over the initial 6 h (*n* = 976).

Variables	All (*n* = 976)	Complete Bundle (*n* = 569)	Incomplete Bundle (*n* = 407)	*p*-Value
6hour total fluid (L), mean (SD)	2.10 ± 1.33	2.34 ± 1.26	1.77 ± 1.36	<0.001
Use of vasopressor, *n* (%)				
Dopamine	50 (5.1)	23 (4.0)	27 (6.6)	0.049
Norepinephrine	969 (99.3)	567 (99.6)	402 (98.8)	0.113
Vasopressin	321 (32.9)	230 (40.4)	91 (22.4)	<0.001
Epinephrine	96 (9.8)	68 (12.0)	28 (6.9)	0.005
Point of care ultrasound, *n* (%)	359 (36.8)	256 (45.0)	103 (25.3)	<0.001
Arterial catheter, *n* (%)	66.1 (67.7)	413 (72.6)	248 (60.9)	<0.001
Central venous catheter, *n* (%)	774 (79.3)	458 (80.5)	316 (77.6)	0.158
Ventilator support, *n* (%)	343 (35.1)	184 (32.3)	159 (39.1)	0.018
Use of inotropic agent, *n* (%)	95 (9.7)	49 (8.6)	46 (11.3)	0.099
Transfusion of RBC, *n* (%)	231 (23.7)	141 (24.8)	90 (22.1)	0.187
Use of corticosteroid therapy, *n* (%)	266 (27.3)	158 (27.8)	108 (26.5)	0.363
Source control, *n* (%)	169 (17.3)	115 (20.2)	54 (13.3)	0.003

Data are presented as *n* (%). ICU and mean ± SD. RBC, red blood cell.

**Table 3:** Clinical outcomes (*n* = 976).

Variables	All (*n* = 976)	Complete Bundle (*n* = 569)	Incomplete Bundle (*n* = 407)	*p*-Value
Transfer to ICU, *n* (%)	578 (59.2)	355 (62.4)	223 (54.8)	0.010
28-day mortality, *n* (%)	429 (44.0)	211 (37.1)	218 (53.6)	<0.001
Hospital mortality, *n* (%)	473 (48.5)	241 (42.4)	232 (57.0)	<0.001

Data are presented as *n* (%). ICU, intensive care unit.

**Table 4:** Multivariate multiple logistic regression model for 28-day mortality in septic shock patients.

Variables	Simple Logistic Regression		Multiple Logistic Regression	
	Unadjusted OR (95% CI)	*p*-Value	Adjusted OR (95% CI)	*p*-Value
Age	1.00 (0.99–1.01)	0.823	1.01 (0.99–1.02)	0.237
Male	0.95 (0.73–1.23)	0.701	1.21 (0.83–1.78)	0.325
Call type (Screening/Direct call)	1.35 (1.04–1.74)	0.022	0.93 (0.63–1.36)	0.691
Complete bundle	0.51 (0.40–0.66)	<0.001	0.61 (0.40–0.91)	0.017
Mechanical ventilator support	2.77 (2.11–3.62)	<0.001	0.88 (0.88–1.43)	0.611
Positive blood culture	0.73 (0.56–0.93)	0.013	0.71 (0.48–1.04)	0.086
Lactate (time zero), mmol/L	1.13 (1.08–1.18)	<0.001	0.93 (0.84–1.03)	0.139
Lactate (re-measurement), mmol/L	1.20 (1.14–1.26)	<0.001	1.20 (1.12–1.29)	<0.001
C-reactive protein, mg/L	1.04 (1.03–1.06)	<0.001	1.04 (1.02–1.06)	<0.001
Procalcitonin, ng/mL	1.00 (1.00–1.00)	0.574	1.00 (1.00–1.00)	0.840
6 h total fluid, L	1.00 (0.91–1.10)	0.960	1.10 (0.53–1.29)	0.200
Arterial catheter	1.35 (1.02–1.77)	0.033	0.59 (0.38–0.91)	0.018
Central venous catheter	1.10 (0.81–1.51)	0.546	0.82 (0.49–1.37)	0.439
Point of care ultrasound	0.84 (0.65–1.09)	0.190	0.99 (0.37–1.45)	0.953
Use of inotropic agent	1.40 (0.92–2.15)	1.404	0.84 (0.45–1.55)	0.569
Transfusion of RBC	1.42 (1.06–1.92)	0.019	1.17 (0.75–1.83)	0.489
Use of corticosteroid therapy	1.49 (1.12–1.98)	0.006	0.89 (0.58–1.39)	0.621
Source control	0.40 (0.28–0.58)	<0.001	0.50 (0.30–0.84)	0.008
SOFA score	1.35 (1.29–1.42)	<0.001	1.33 (1.23–1.44)	<0.001

OR, odds ratio, CI, confidence interval, RBC, red blood cell, SOFA, sequential (sepsis-related) organ failure assessment.

## Data Availability

Not applicable.

## Supplementary Materials

The following are available online at https://www.mdpi.com/article/10.3390/jcm10184244/s1, Table S1. Sepsis bundle compliance and 28-day mortality for patients with septic shock over 10 years, Table S2. Clinical characteristics of the 976 patients.
