# Supplementary material for: Rapid Response System Improves Sepsis Bundle Compliances and Survival in Hospital Wards for 10 Years
Source: J Clin Med. 2021 Sep 18;10(18):4244. doi: 10.3390/jcm10184244 (PMC8466148; doi:10.3390/jcm10184244)
Supplement: Supplementary file 1 [file jcm-10-04244-s001.zip › jcm-1366573-Table S1.pdf]

Table S1. Sepsis bundle compliance and 28-day mortality for patients with septic shock over 10 years

Variables	All (*n* = 976)	2008 (*n* = 34)	2009 (*n* = 65)	2010 (*n* = 113)	2011 (*n* = 91)	2012 (*n* = 106)	2013 (*n* = 95)	2014 (*n* = 101)	2015 (*n* = 121)	2016 (*n* = 110)	2017 (*n* = 140)	*p*-Value
Measurement of lactate/3hour	939 (96.2)	33 (97.1)	57 (87.7)	99 (87.6)	88 (96.7)	104 (98.1)	94 (98.9)	101 (100.0)	119 (98.3)	107 (97.3)	137 (97.9)	<0.001
Obtain blood cultures/3hour	791 (81.0)	20 (58.8)	43 (66.2)	81 (71.7)	73 (80.2)	85 (80.2)	77 (81.1)	84 (83.2)	107 (88.4)	97 (88.2)	124 (88.6)	<0.001
Administer broad-spectrum intravenous antibiotics/3hour	940 (96.3)	32 (94.1)	62 (95.4)	104 (92.0)	85 (93.4)	104 (98.1)	91 (95.8)	100 (99.0)	118 (97.5)	107 (97.3)	137 (97.9)	0.144
Administer 30 mL/kg crystalloid/3hour	885 (90.7)	28 (82.4)	64 (98.5)	101 (89.4)	87 (95.6)	94 (88.7)	86 (90.5)	93 (92.1)	108 (89.3)	100 (90.9)	124 (88.6)	0.210
Application of vasopressors/6hour	976 (100.0)	34 (100.0)	65 (100.0)	113 (100.0)	91 (100.0)	106 (100.0)	95 (100.0)	101 (100.0)	121 (100.0)	110 (100.0)	140 (100.0)	
Repeat the lactate measurement/6hour	789 (80.8)	16 (47.1)	39 (60.0)	73 (64.6)	72 (79.1)	92 (86.8)	86 (90.5)	85 (84.2)	102 (84.3)	98 (89.1)	126 (90.0)	<0.001
Complete bundle. overall	569 (58.3)	9 (26.5)	26 (40.0)	39 (34.5)	49 (53.8)	65 (61.3)	61 (64.2)	66 (65.3)	81 (66.9)	75 (68.2)	98 (70.0)	<0.001
28-day mortality	429 (44.0)	17 (50.0)	30 (46.2)	51 (45.1)	49 (53.8)	48 (45.3)	44 (46.3)	56 (55.4)	47 (38.8)	42 (38.2)	45 (32.1)	0.014

Data are presented as *n* (%).
