# Supplementary material for: Rapid Response System Improves Sepsis Bundle Compliances and Survival in Hospital Wards for 10 Years
Source: J Clin Med. 2021 Sep 18;10(18):4244. doi: 10.3390/jcm10184244 (PMC8466148; doi:10.3390/jcm10184244)
Supplement: Supplementary file 1 [file jcm-10-04244-s001.zip › jcm-1366573-Table S2.pdf]

**Table S2.** Clinical characteristics of the 976 patients

| Variables | All (*n* = 976) | Complete Bundle (*n* = 569) | Incomplete Bundle (*n* = 407) | *p*-Value |
|---|---|---|---|---|
| Age, years, mean (SD) | 61.5 ± 13.5 | 61.9 ± 13.1 | 60.9 ± 14.0 | 0.284 |
| Male, *n* (%) | 594 (60.9) | 349 (61.3) | 245 (60.2) | 0.384 |
| SOFA score, mean (SD) | 10.8 ± 3.5 | 10.6 ± 3.3 | 11.1 ± 3.7 | 0.029 |
| Co-morbidity, *n* (%) | | | | |
| Solid tumor | 421 (43.1) | 255 (44.8) | 166 (40.8) | 0.117 |
| Hematologic malignancy | 299 (30.6) | 176 (30.9) | 123 (30.2) | 0.434 |
| Chronic lung disease | 51 (5.2) | 27 (4.7) | 24 (5.9) | 0.256 |
| Chronic heart disease | 168 (17.2) | 100 (17.6) | 68 (16.7) | 0.396 |
| Chronic liver disease | 152 (15.6) | 88 (15.5) | 64 (15.7) | 0.490 |
| Chronic renal disease | 64 (6.6) | 34 (6.0) | 30 (7.4) | 0.230 |
| DM | 277 (28.4) | 173 (30.4) | 104 (25.6) | 0.056 |
| Department, *n* (%) | | | | |
| Medical | 876 (89.8) | 509 (89.5) | 367 (90.2) | 0.595 |
| Surgical | 97 (9.9) | 59 (10.4) | 38 (9.3) | |
| Maternal | 3 (0.3) | 1 (0.2) | 2 (0.2) | |
| Type of activation, *n* (%) | | | | |
| Screening | 561 (57.5) | 342 (60.1) | 219 (53.8) | 0.029 |
| Direct call | 415 (42.5) | 227 (39.9) | 188 (46.2) | |
| Source of infection, *n* (%) | | | | |
| Intra-abdominal | 434 (44.5) | 275 (48.3) | 159 (39.1) | 0.037 |
| Pneumonia | 261 (26.7) | 131 (23.0) | 130 (31.9) | |
| Bacteremia | 134 (13.7) | 77 (13.5) | 57 (14.0) | |
| Urinary tract infection | 58 (5.9) | 35 (6.2) | 23 (5.7) | |
| Other | 64 (6.6) | 37 (6.5) | 27 (6.6) | |
| Unknown | 25 (2.6) | 14 (2.5) | 11 (2.7) | |
| Blood culture, *n* (%) | | | | |
| Positive; Gram-positive | 79 (8.7) | 44 (7.8) | 35 (10.3) | 0.003 |
| Positive; Gram-negative | 317 (35.1) | 223 (39.7) | 94 (27.6) | |
| Positive; Poly-microbial | 65 (7.2) | 43 (7.7) | 22 (6.5) | |
| Positive; Fungus | 13 (1.4) | 7 (1.2) | 6 (1.8) | |
| Negative | 429 (47.5) | 245 (43.6) | 184 (54.0) | |
| Laboratory findings, mean (SD) | | | | |
| Platelets, X$10^3$/L | 114.72 ± 109.92 | 116.80 ± 112.65 | 119.72 ± 116.44 | 0.495 |

| | | | | |
|---|---|---|---|---|
| C-reactive protein, mg/L | 11.38 ± 8.80 | 12.20 ± 9.52 | 13.38 ± 10.37 | 0.002 |
| Procalcitonin, ng/mL | 33.20 ± 70.01 | 30.28 ± 63.83 | 25.20 ± 51.05 | 0.068 |
| Creatinine, mg/dL | 1.57 ± 1.09 | 1.68 ± 1.32 | 1.85 ± 1.59 | 0.002 |
| Bilirubin, mg/dL | 4.01 ± 6.64 | 4.12 ± 6.74 | 4.29 ± 6.89 | 0.523 |
| Lactate (time zero), mmol/L | 5.25 ± 3.00 | 5.12 ± 3.06 | 4.92 ± 3.14 | 0.102 |
| Lactate (re-measurement), mmol/L | 4.74 ± 3.04 | 4.78 ± 3.19 | 4.94 ± 3.50 | 0.340 |

Data are presented as *n* (%) and mean ± SD. SOFA, sequential (sepsis-related) organ failure assessment.
